# An Observational Study to Develop a Predictive Model for Bacterial Pneumonia Diagnosis in Severe COVID-19 Patients—C19-PNEUMOSCORE

**DOI:** 10.3390/jcm12144688

**Published:** 2023-07-14

**Authors:** Eloisa Sofia Tanzarella, Joel Vargas, Marco Menghini, Stefania Postorino, Francesca Pozzana, Maria Sole Vallecoccia, Francesco Lorenzo De Matteis, Federico Franchi, Amato Infante, Luigi Larosa, Maria Antonietta Mazzei, Salvatore Lucio Cutuli, Domenico Luca Grieco, Alessandra Bisanti, Simone Carelli, Gianmarco Lombardi, Edoardo Piervincenzi, Gabriele Pintaudi, Tommaso Pirronti, Mario Tumbarello, Massimo Antonelli, Gennaro De Pascale

**Affiliations:** 1 Dipartimento di Scienze Dell’emergenza, Anestesiologiche e della Rianimazione, Fondazione Policlinico Universitario A. Gemelli IRCCS, 00168 Rome, Italy; eloisasofia.tanzarella@policlinicogemelli.it (E.S.T.); stefania.postorino@policlinicogemelli.it (S.P.); salvatorelucio.cutuli@policlinicogemelli.it (S.L.C.); domenicoluca.grieco@policlinicogemelli.it (D.L.G.); alessandra.bisanti@policlinicogemelli.it (A.B.); simone.carelli@policlinicogemelli.it (S.C.); gianmarco.lombardi@policlinicogemelli.it (G.L.); edoardo.piervincenzi@policlinicogemelli.it (E.P.); gabriele.pintaudi@policlinicogemelli.it (G.P.); massimo.antonelli@policlinicogemelli.it (M.A.); 2 Dipartimento di Scienze Cardiovascolari, Fondazione Policlinico Universitario A. Gemelli IRCCS, 00168 Rome, Italy; joel.vargas@policlinicogemelli.it; 3 U.O.C. Terapia Intensiva OM e Hub Maxi Emergenze, Ospedale Maggiore Carlo Alberto Pizzardi, 40133 Bologna, Italy; m.menghini@hotmail.it; 4 Dipartimento di Anestesia e Rianimazione, Ospedale Santa Maria Goretti, 04100 Latina, Italy; francesca.pozzana@gmail.com; 5 Anesthesia and Intensive Care Unit, Department of Emergency and Critical Care, Santa Maria Nuova Hospital, 50122 Florence, Italy; mariasole.vallecoccia@gmail.com; 6 Department of Medical Science, Surgery and Neurosciences, Cardiothoracic and Vascular Anesthesia and Intensive Care Unit, University of Siena, 53100 Siena, Italy; francesco.dematteis@dbm.unisi.it (F.L.D.M.); federico.franchi@dbm.unisi.it (F.F.); 7 Dipartimento di Diagnostica per Immagini, Radioterapia Oncologica ed Ematologia, Fondazione Policlinico Universitario A. Gemelli IRCCS, 00168 Rome, Italy; amato.infante@policlinicogemelli.it (A.I.); luigi.larosa@policlinicogemelli.it (L.L.); tommaso.pirronti@policlinicogemelli.it (T.P.); 8 Unit of Diagnostic Imaging, Department of Medical, Surgical and Neuro Sciences and of Radiological Sciences, University of Siena, Azienda Ospedaliero-Universitaria Senese, 53100 Siena, Italy; mariaantonietta.mazzei@unisi.it; 9 Dipartimento di Biotecnologie Mediche, Università degli Studi di Siena, 53100 Siena, Italy; mario.tumbarello@unisi.it

**Keywords:** bacterial co-infection, COVID-19, bacterial pneumonia, prognostic tool, antimicrobial stewardship

## Abstract

In COVID-19 patients, antibiotics overuse is still an issue. A predictive scoring model for the diagnosis of bacterial pneumonia at intensive care unit (ICU) admission would be a useful stewardship tool. We performed a multicenter observational study including 331 COVID-19 patients requiring invasive mechanical ventilation at ICU admission; 179 patients with bacterial pneumonia; and 152 displaying negative lower-respiratory samplings. A multivariable logistic regression model was built to identify predictors of pulmonary co-infections, and a composite risk score was developed using β-coefficients. We identified seven variables as predictors of bacterial pneumonia: vaccination status (OR 7.01; 95% CI, 1.73–28.39); chronic kidney disease (OR 3.16; 95% CI, 1.15–8.71); pre-ICU hospital length of stay ≥ 5 days (OR 1.94; 95% CI, 1.11–3.4); neutrophils ≥ 9.41 × 10^9^/L (OR 1.96; 95% CI, 1.16–3.30); procalcitonin ≥ 0.2 ng/mL (OR 5.09; 95% CI, 2.93–8.84); C-reactive protein ≥ 107.6 mg/L (OR 1.99; 95% CI, 1.15–3.46); and Brixia chest X-ray score ≥ 9 (OR 2.03; 95% CI, 1.19–3.45). A predictive score (C19-PNEUMOSCORE), ranging from 0 to 9, was obtained by assigning one point to each variable, except from procalcitonin and vaccine status, which gained two points each. At a cut-off of ≥3, the model exhibited a sensitivity, specificity, positive predictive value, negative predictive value, and accuracy of 84.9%, 55.9%, 69.4%, 75.9%, and 71.6%, respectively. C19-PNEUMOSCORE may be an easy-to-use bedside composite tool for the early identification of severe COVID-19 patients with pulmonary bacterial co-infection at ICU admission. Its implementation may help clinicians to optimize antibiotics administration in this setting.

## 1. Introduction

At the end of 2019, severe acute respiratory syndrome coronavirus 2 (SARS-CoV-2) emerged as the cause of coronavirus disease 2019 (COVID-19). Since then, many variants, different in terms of virulence and transmissibility, have been reported, resulting in more than 6.8 million deaths globally. Currently, despite the extraordinary impact of vaccination programs on the course of the COVID-19 pandemic, certain populations are still at risk of developing life-threatening complications of the disease [1]. In fact, the immune response to SARS-CoV-2 vaccines of patients with primary or secondary immunodeficiency may be significantly impaired, being associated with prolonged hospitalization, nosocomial complications, and high mortality rates [2].

Acute respiratory distress syndrome (ARDS), as the most frequent life-threatening complication of COVID-19, is typically associated with hyper-inflammatory syndrome that may resemble clinical patterns of common bacterial infections [3,4]. Indeed, at intensive care unit (ICU) admission, understanding which patients with severe COVID-19 are deteriorating due to the overwhelming immune disorder or to concomitant bacterial pneumonia is a real challenge for clinicians. The reported incidence rate of bacterial co-infections among hospitalized COVID-19 patients is quite low, ranging from 5% in non-critically ill settings [5,6] up to 28% in ICUs [7,8,9]. Despite that, broad-spectrum empiric antimicrobial therapy is widely prescribed [10], especially in the intensive care setting where the severity of clinical features and the poor outcomes of such critically ill patients often lead physicians to administer antibiotics without evidence of bacterial infection [11].

Therefore, prompt recognition of the most common risk factors for bacterial co-infection at ICU admission may be of paramount importance, with the aim of avoiding inappropriate use of empiric therapy in patients with only viral pneumonia, without missing those really needing antibiotics.

Although some authors have already identified main risk factors for bacterial concomitant infections in COVID-19 patients, the majority of them have focused their attention on the non-critically ill setting, including diseases other than pneumonia (e.g., bloodstream and urinary tract infections) [7,12]. Conversely, in the ICU, few data are currently available, mainly describing clinical features and outcomes compared with other viral pneumonia (i.e., influenza) [13,14].

In light of this, we decided to analyze epidemiological, clinical, laboratory, and radiological features of a large cohort of severe COVID-19 patients undergoing invasive mechanical ventilation within 48 h from ICU admission. The objective was to create an easy-to-use bedside composite predictive model to identify concomitant bacterial pneumonia at ICU admission in patients with COVID-19 ARDS.

## 2. Methods

### 2.1. Study Setting and Design

**Figure 1:**
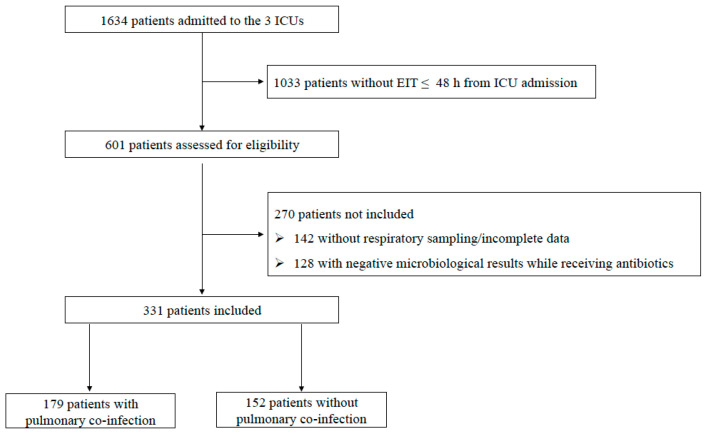
Flow chart of the study inclusion process. Legend: EIT: endotracheal intubation; ICU: intensive care unit.

This multicenter observational study included 331 patients with severe COVID-19, hospitalized across three university hospital ICUs in Italy (Fondazione Policlinico Universitario A. Gemelli IRCCS, Rome; Azienda Ospedaliera Universitaria Senese, Siena; Ospedale Santa Maria Nuova, Firenze) between March 2020 and June 2022. All patients ≥18 years old requiring endotracheal intubation (ETI) and invasive mechanical ventilation (IMV) within 48 h of ICU admission were assessed for eligibility and underwent deep respiratory sampling for microbiological examination (either tracheal aspirate [TA] or bronchoalveolar lavage [BAL]), two sets of blood cultures, and *Legionella* urinary antigen tests. Exclusion criteria included the following: ETI performed >48 h from ICU admission; unconfirmed SARS-CoV-2 infection; lack of respiratory samples; and incomplete data. Patients with negative microbiological results while receiving antibiotics in the previous 15 days were also excluded as they were considered potential false negatives (Figure 1). Electronic patient records (Digistat^®^) and microbiology laboratory data (TrakCare^®^) were used to identify patients and to retrieve clinical and microbiological results. These data included demographic characteristics (age, sex, vaccination status, length of symptoms and hospitalization); main comorbidities, clinical and laboratory findings; main severity scores (Charlson Comorbidity Index (CCI) [15], Simplified Acute Physiology Score II (SAPS II) [16], and Sequential Organ Failure Assessment (SOFA) score [17]); radiological features on chest-X-ray; on-going treatments (antibiotics type, use of steroids, antivirals, and immunomulatory agents); and outcome measures (hospital/ICU length of stay, need of tracheostomy, and 28-day/90-day mortality). The study was performed in accordance with the Declaration of Helsinki and was approved by the coordinator center ethics committee (FPG-UCSC reference number ID3141). Written informed consent or proxy consent was waived due to observational nature of the study, according to committee recommendations. All data were anonymous and were identified with an admission code number.

Reporting of the study followed the “Strengthening the Reporting of Observational Studies in Epidemiology” (STROBE) guidelines for observational studies [18].

### 2.2. Definitions and Outcomes

Concomitant bacterial pneumonia was defined as a positive lower-respiratory-tract sampling performed soon after endotracheal intubation, according to 10^5^ CFU/mL and 10^4^ CFU/mL thresholds for TA and BAL, respectively. The episode was defined as bacteremic when the pathogen retrieved from BAL/TA was also isolated in at least one blood culture, in the absence of another specified source of bacteremia [19,20]

Radiological findings were classified according to a multi-valued scoring system, i.e., the “Brixia score”. Chest-X-rays on ETI day were used to compute the score; the two lungs were divided into six regions, and each region was assigned an integer rating from 0 to 3 based on the local assessed severity of parenchymal impairment [21]. All chest-X-rays were independently evaluated by two senior radiologists (AI and LLR) who were blind to the microbiological diagnosis. The judgement of the two radiologists was not unanimous in less than 10% of cases. When this occurred, they both reassessed the images and reached a consensus decision. The primary outcome of this study was to develop an easy-to-use bedside composite score for COVID-19 concomitant bacterial pneumonia diagnosis at ICU admission, including epidemiological, clinical, laboratory, and radiological features. The secondary objectives were the description of microbiological findings, antibiotic therapies, and main outcomes according to concomitant bacterial pneumonia diagnosis.

### 2.3. Statistical Analysis

The Kolmogorov–Smirnov test was used to evaluate the distribution of variables. Data with a non-normal distribution were assessed using the Mann–Whitney test, and the median and selected centile (25th–75th) values are given. The data with a normal distribution were assessed using Student’s *t* test. Categorical variables are given as proportions and were analyzed using the chi-squared test or Fisher’s exact test, as appropriate. *p* < 0.05 was considered significant. The crude odds ratio (OR) and 95% CI were calculated for each variable. We included all variables in the multivariable logistic regression if they reached *p* ≤ 0.1 during univariate analysis. A stepwise selection procedure was used to select variables for inclusion in the final model. Overall goodness of fit was analyzed using Nagelkerke’s R-square. Discrimination of the model was assessed by receiver operating characteristic (ROC) curve characteristics. To develop the risk score, variables that maintained statistical significance in the multivariate regression model were assigned a point value corresponding to the β-coefficient (fixed effects) rounded to the nearest integer. Summation of the points generated by the calculated risk factors resulted in a quantitative score (from 0 to 9) that was assigned to each patient in the database. All statistical analyses were performed using SPSS Statistical Software version 28.0.1.0 (IBM Corporation, Armonk, NY, USA), whereas data were graphed using GraphPad Prism version 6.0 (GraphPad Software, San Diego, CA, USA).

### 2.4. Microbiological Analysis

Nasopharyngeal swabs were obtained from COVID-19 patients to detect one or more SARS-CoV-2-specific nucleic acid targets by the Korean Ministry of Food and Drug Safety-approved Allplex™ 2019-nCoV assay (Arrow Diagnostics S.r.l., Genova, Italy), which is a real-time reverse-transcriptase–polymerase-chain-reaction (RT-PCR)-based assay for SARS-CoV-2 RNA detection [22]. A positive RT-PCR result was used to confirm COVID-19 diagnosis, which in turn relied on the presence of fever and/or lower-respiratory-tract symptoms and on lung imaging features consistent with SARS-CoV-2 pneumonia. The TA/BAL fluid samples were immediately sent to the microbiology laboratory of each hospital for microbiological investigations, consisting of Gram staining examination and (qualitative or quantitative) aerobic cultures on standard agar media. For microbial isolates, species identification was performed using the MALDI Biotyper system (Bruker Daltonics, Bremen, Germany), and in vitro antimicrobial susceptibility testing was performed using Vitek 2 (bioMérieux, Mercy l’Étoile, France) or MERLIN Diagnostica GmbH (Bornheim, Germany) broth microdilution systems. Minimum inhibitory concentrations were interpreted in accordance with the European Committee on Antimicrobial Susceptibility Testing (EUCAST) clinical breakpoints. In the coordinating center (Fondazione Policlinico Universitario ‘A. Gemelli’ IRCCS), fast microbiology results from respiratory samples were also provided (FilmArray Pneumonia Panel Plus [BioFire, Salt Lake City, UT, USA]) [23].

## 3. Results

### 3.1. Population Characteristics, Treatments and Outcomes

During the study period, 1634 COVID-19 patients were admitted to three ICUs; 1303 were excluded because they were not intubated within first 48 h, did not undergo respiratory sampling, showed negative microbiological results while receiving antibiotics, or because complete data were not available. Of the 331 patients included in the analysis, 179 were classified as concomitant bacterial pneumonia and 152 were classified as viral-only lung respiratory infections (Figure 1).

Overall population median SAPS II, SOFA, and CCI scores were 39, 5, and 3, respectively, and the main comorbidities were represented by obesity (29%), diabetes (26.9%), and chronic heart disease (CHD) (20.8%) with a median pre-ICU hospital length of stay of 5 days (Table 1). Almost 20% of the patients were already colonized with multidrug-resistant (MDR) bacteria at ICU admission. On chest X-ray evaluation, the median total Brixia score was 9, and the rate of consolidations and pleural effusion (either mono or bilateral) ranged between 10.9% and 34.4%. About 85% and 50% of the patients were treated with dexamethasone and remdesivir at ICU admission, respectively, and in only 44 cases (13.3%), IL-6 inhibitors were already administered. Mortality rates at 28 days and 90 days were 43.5% and 50.3%, respectively, with pretty long median durations of ICU and hospital stay (15 and 20 days) (Table 1).

As per the protocol definition, no patients classified as only SARS-CoV-2 pneumonia were receiving antibiotics or had positive TA/BAL at ICU admission. A descriptive analysis of the microbiological features and antimicrobial therapies of patients with pulmonary bacterial co-infection is reported in Table 2. Among the 179 co-infected patients, 60 (33.5%) were receiving empiric antimicrobial therapy at ICU admission, namely cephalosporins (11.7%), beta lactamase + inhibitors (15.1%), and macrolides (10.6%), above all. We observed rates of polymicrobial and MDR infections of 23.5% and 20.1%, respectively, with 32 patients (17.9%) showing concomitant bloodstream infection. We did not observe prevalence between Gram-positive and Gram-negative pathogens; the former group was mainly represented by *Staphylococcus aureus* (44.7%) and *Streptococcus pneumoniae* (7.8%), while the latter was mainly represented by *Pseudomonas aeruginosa* (17.3%), *Klebsiella pneumoniae* (13.9%), and *Haemophilus influenzae* (7.8%).

### 3.2. Factors Associated with Bacterial Pulmonary Co-Infection and Score Development

ICU-admission descriptive characteristics of patients with and without pulmonary bacterial co-infection are shown in Table 1, including epidemiological, clinical, laboratory, and radiology-presenting features.

Following univariate analysis, the predictors of bacterial pneumonia were as follows: COVID-19 vaccination status; recent hospitalization; obesity; CHD; non-cardiac vasculopathy; chronic kidney disease (CKD); CCI, SAPS II, and SOFA score; pre-ICU length of stay; MDR colonization; neutrophils count; procalcitonin (PCT) and C-reactive protein (CRP) values; and total Brixia score.

Multivariable logistic regression confirmed only seven of these variables as independent risk factors for ICU-admission bacterial pulmonary co-infection: vaccination status (OR 7.01; 95% CI, 1.73–28.39); chronic kidney disease (OR 3.16; 95% CI, 1.15–8.71); pre-ICU hospital LOS ≥ 5 days (OR 1.94; 95% CI, 1.11–3.4); neutrophils ≥ 9.4 × 10^9^/L (OR 1.96; 95% CI, 1.16–3.30); PCT ≥ 0.2 ng/mL (OR 5.09; 95% CI, 2.93–8.84); CRP ≥ 108 mg/L (OR 1.99, 95% CI, 1.15–3.46); and Brixia chest X-ray score ≥ 9 (OR 2.03; 95% CI, 1.19–3.45).

**Figure 2:**
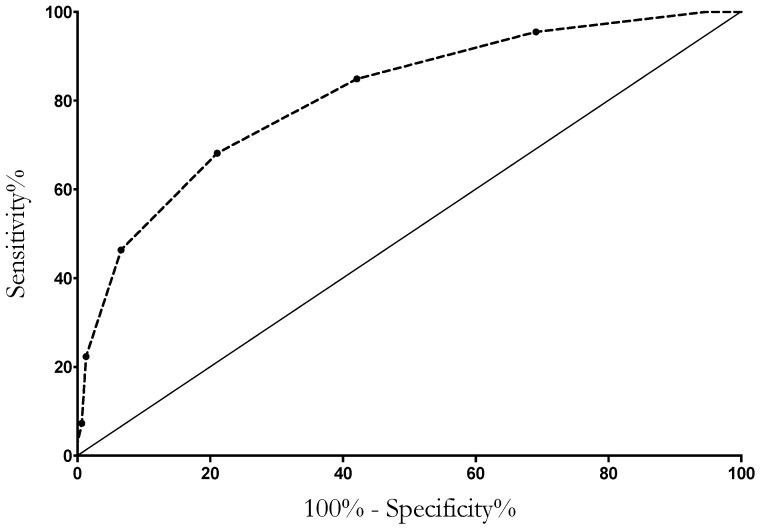
Discrimination of co-infection status using area under the ROC curve analysis. Area under ROC curve: 0.81 (0.76–0.86).

For each variable, a point was assigned according to the β-coefficient rounded up to the nearest integer. The predictive score resulted from the sum of the individual points (Table 3). Discrimination of co-infection status was performed using the area under the ROC curve analysis, with a result of 0.81 (0.76–0.86) (Figure 2). An individual predictive score (C19-PNEUMOSCORE), ranging from 0 to 9, was obtained by assigning one point to each variable, except for procalcitonin and vaccine status, which gained two points each. At a cut-off of ≥3, the model exhibited a sensitivity, specificity, positive predictive value, negative predictive value, and accuracy of 84.9%, 55.9%, 69.4%, 75.9%, and 71.6%, respectively (Table 4).

## 4. Discussion

In this multicenter observational study of 331 patients with severe COVID-19 acute respiratory failure (ARF), the rate of concomitant bacterial pneumonia at ICU admission was 25.3%. By analyzing epidemiological, clinical, laboratory, and radiological features, we developed an easy-to-use bedside score (C19-PNEUMOSCORE) to identify patients at high risk of bacterial lung co-infection. This score may be useful for ICU clinicians to individualize the need for antibiotic therapy in patients with severe COVID-19, especially when diagnostic sampling and fast microbiology are not promptly available.

The rate of concomitant bacterial pneumonia in critically ill patients with COVID-19 is variable, mainly depending on the severity of pneumonia and hospitalization length [24,25]. In a large multicenter study, Rouzé et al. observed that only 9.7% of 568 patients performing a BAL within 48 h of intubation had bacterial isolation [14], compared with 33.6% of those with severe influenza. In our cohort of 601 eligible patients, after excluding cases without deep respiratory samplings or possible false-negative results (Figure 1), 179 (29.8%) had a bacterial lung co-infection; such a rate might be interpreted in light of the high severity of our study population (median P/F ratio was 88, with a mortality rate of 43.5%) and the frequent use of rapid multiplex PCR for bacterial identification. Similarly, in a cohort of intubated patients with severe COVID-19, the percentage of bacterial identification from deep respiratory samples increased from 17.3% to 24.5% when a molecular diagnostic assay was added to the standard cultures [26].

Despite the knowledge that less than one-third of deteriorating patients with severe COVID-19 has bacterial pneumonia, the use of empirical antibiotics has been largely diffused, contributing to the increase in the rate of secondary MDR nosocomial infections in the ICU setting [27]. In a large international cohort study involving 4994 patients, the rate of bacterial co-infection at ICU admission was 14%, whilst the percentage of empirical antibiotic therapy was 85% [28] The authors documented 2715 (54%) cases of nosocomial infections, mainly represented by ventilator-associated pneumonia (44%) with an MDR rate of 25%. In light of that, antimicrobial stewardship programs in the ICU setting have been strongly advocated for, with the aim of reducing indiscriminate antibiotic exposure in such patient categories, without missing ones with concomitant bacterial infection [29]. Interestingly, in our cohort, 188 of 601 (31.3%) patients were receiving antibiotics at ICU admission: 128 with subsequent negative microbiological results and 60 with bacterial pneumonia. Such a low percentage of patients receiving antibiotics can be explained by the application of antimicrobial stewardship programs in the pre-ICU settings of the three hospitals, especially during the second and third wave of the COVID-19 pandemic. Once in the ICU, a respiratory sampling was promptly performed, and watchful waiting was encouraged by fast microbiology, which yielded reliable results in less than 3 h. Therefore, although some of our patients probably received antimicrobials without evidence of infection, it is possible that another part was not promptly identified as co-infected, thus delaying appropriate treatment.

Indeed, a multimodal approach including risk factors, presenting features, biomarkers levels, and early microbiological results could help clinicians discriminate between those who do not need antibiotics and those where empirical therapy is warranted [30].

Although predictors of bacterial pulmonary co-infections have been described in the general population, data in the critically ill setting are actually lacking, especially in patients admitted to ICUs while receiving non-invasive respiratory support, where lung sampling is not simply to be performed [12,31].

In our population, we identified seven independent risk factors for bacterial co-infection at ICU admission, and some of them are of particular clinical interest. We observed that the longer the length of hospital stay, the higher the probability of isolating bacteria, with an MDR rate of 20.1%. This information is biologically sound since hospitalization and healthcare assistance are key drivers for respiratory microbiome changes, which may increase the risk of virulent agent proliferation in the tracheobronchial tree [32].

Interestingly, fully vaccinated patients admitted to the ICU showed a seven-fold higher odd of having bacterial pneumonia; such a risk profile may be interpreted in light of the phenotype of critically ill, COVID-19, vaccinated patients who are usually immunosuppressed, older, and with several comorbidities, all of which are well-known risk factors for bacterial pneumonia [33]. This statement is often reported in the current literature and seems to be the most “epidemiologically sound” sentiment, but it does not seem to be confirmed by our data. However, there is another issue worth considering. Since vaccination programs have radically changed the natural history of COVID-19, the disease has become increasingly less harmful in vaccinated patients. Therefore, we can assume that if one of those vaccinated patients develops pneumonia severe enough to be admitted to the ICU, maybe a bacterial co-infection should be suspected to justify the severity of symptoms.

Similarly, the association of CKD with an increased risk of bacterial co-infection is not surprising, as these patients have a complex defect of almost all components of the immune system and are more likely to develop severe COVID-19 pneumonia [34,35].

Again, we identified that high neutrophil count (≥9.4 × 10^9^/L), PCT (≥0.2 ng/mL), and CRP (≥108 mg/L) were all independent predictors of co-infection at ICU admission. These results are consistent with the most recent literature, both in critically ill patients and the less severe population. In a large observational study of 4635 patients from 84 ICUs, neither PCT nor CRP were independently associated with bacterial co-infection, but baseline values of PCT < 0.3 ng/mL could be useful to rule out bacterial pneumonia (negative predictive value of 91.1%) [36]. Similarly, in the early phase of the pandemic, the prognostic value of commonly used biomarkers (PCT, CRP, and neutrophils) was investigated in 298 patients with severe COVID-19 [37]. The authors observed that CRP, with a cut-off value of 41.4 mg/L, had the highest diagnostic accuracy (AUC 0.896), with a sensitivity of 90.5%, specificity of 77.6%, positive predictive value of 61.3%, and negative predictive value of 95.4%; it was also an independent predictor of ICU admission. Similarly, in another recent large observational study, the only predictors of bacterial pneumonia were previous hospitalization, severity of illness, and leukocytosis [12]. In addition, combined use of both PCT and CRP may further improve the capability to predict bacterial pneumonia early. In a cohort of 224 COVID-19 patients, elevated PCT was associated with a higher likelihood of co-infection and death, and low CRP levels were strongly predictive of low PCT concentrations with a negative predictive value of almost 100% [38]. However, despite the fact that the above data would suggest the usefulness of a biomarker-based stewardship program in COVID-19, the only randomized trial addressing such an issue, failed to show any benefit [39]. Randomizing patients to standard antibiotic prescription or to a protocol including daily PCT (cut-off for antibiotics of 1ng/mL) and multiplex PCR respiratory diagnostic, no differences in terms of antibiotic consumption and clinical outcomes were observed. This may be due to the more complex picture of critically ill patients with COVID-19, where mere biomarker evaluation cannot be enough to best manage antimicrobial prescriptions, and a multimodal approach, including radiological features, should be preferred.

Indeed, in our study, we observed that a Brixia score ≥ 9 predicted bacterial co-infection, although without any correlation with the distribution of consolidations nor even the presence of pleural effusion. This radiological score was developed in order to interpret chest X-ray alterations as an indicator of the extent of the changes in lung parenchyma, and it was validated as a radiological predictive tool for invasive mechanical ventilation and mortality [19]. It has been said that it is not surprising that it may be associated with a higher probability of bacterial lung co-infection, as it is mainly driven by the presence of consolidative opacities and multi-lobar involvement.

Given all the above considerations, we decided to elaborate a composite score, C19-PNEUMOSCORE, which merges different contributing factors for co-infection development, identifying a cut-off value of 3 as the best value to balance the risk of antibiotic over- and under-treatment. To the best of our knowledge, there is only one other study where a bedside score was developed [7], but it has many differences to ours. In this paper, Giannella et al., after identifying white blood cells, PCT, and the Charlson Index as independent predictors, built three risk categories (low, intermediate, and high), according to the score and CURB-65 value. Differently from our cohort, this investigation was not performed in the ICU setting, did not include only patients with pneumonia (42%), and did not investigate the role of radiological findings.

We acknowledge several limitations for this study: firstly, there was a lack of a validation cohort that could confirm the strength of our observations. Otherwise, it was also clear that the application of C19-PNEMOSCORE in an interventional study, by treating patients on spontaneous breathing with values ≥ 3, could not yield the correct interpretation of microbiological findings from invasive respiratory sampling in the case of endotracheal intubation. Secondly, we only considered SARS-CoV-2 infection; indeed, we cannot extrapolate that the score may also be effective in viral infections other than COVID-19. Furthermore, among 601 patients, 270 were excluded due to incomplete data or negative microbiological results while receiving antibiotics. Although we are aware that this could lead to selection bias, we could not consider patients with negative respiratory sampling while on antimicrobial therapy because it was not possible to rule out the possibility that they only had SARS-CoV-2 pneumonia without bacterial co-infection. On the other hand, all 142 patients with incomplete data had either clear contraindications to safely perform a Bal with an inadequate amount of secretions to perform a tracheal aspirate instead, or they lacked of one of the seven variables identified as predictors of bacterial pneumonia in the data collection.

Finally, enrolling ICU patients independently from the length of previous hospitalization, we obviously included not only community-acquired co-infections, but also non-ICU nosocomial bacterial co-infections. Although this aspect may represent a limitation, it actually does not change the aim of the study, which was to focus on the prompt identification of patients requiring antibiotics at ICU admission.

However, this is the first study where an easy-to use, bedside score has been developed as a tool to stratify the risk of bacterial co-infection and to optimize antibiotic use in COVID-19, critically ill, deteriorating patients, including those who already underwent complete vaccination.

## 5. Conclusions

A composite, easy-to-use, bedside score (C19-PNEUMOSCORE) including presenting features (vaccination status, pre-ICU length of stay, chronic kidney disease), laboratory parameters (neutrophils, PCT, CRP), and chest X-ray evaluation (Brixia score) may be a useful tool for the prompt stratification of patients with bacterial co-infection. The application of such a score may become part of antibiotic stewardship programs in the management of COVID-19 patients, including those already vaccinated, especially when deep respiratory sampling is not available.

## Figures and Tables

**Table 1:** Univariate analysis of factors associated with bacterial pulmonary co-infection at ICU admission.

Variables	No. of Patients	No. of Patients		Univariate Analysis	
	Total Cohort (n = 331)	No Co-Infection (n = 152)	Co-Infection (n = 179)	*p* Value	OR (95% CI)
*Demographics and comorbidities*					
Age, years	67 [59–74]	65 [58–72]	68.5 [60–74]	0.23	1.01 (0.99–1.03)
Gender (male)	233 (70.4)	101 (66.45)	132 (73.74)	0.15	1.42 (0.88–2.28)
COVID-19 Vaccinated	24 (7.3)	3 (2)	21 (11.73)	0.003	6.6 (1.93–22.59)
Recent hospitalization *	29 (8.8)	8 (5.3)	21 (11.7)	0.04	2.39 (1.03–5.57)
BMI ≥ 30 kg/m^2^	96 (29.0)	55 (36.2)	41 (22.9)	0.008	0.52 (0.32–0.85)
CHD **	69 (20.8)	20 (13.2)	49 (27.4)	0.002	2.49 (1.40–4.42)
COPD	70 (21.1)	29 (19.1)	41 (22.9)	0.39	1.26 (0.74–2.15)
Non-cardiac vasculopathy	45 (13.6)	13 (8.6)	32 (17.9)	0.02	2.33 (1.17–4.62)
Diabetes	89 (26.9)	34 (22.4)	55 (30.7)	0.09	1.54 (0.94–2.53)
CKD	41 (12.4)	7 (4.6)	34 (19.0)	<0.001	4.86 (2.09–11.31)
Immunosuppression ***	60 (18.0)	26 (17.0)	34 (19.0)	0.51	0.83 (0.47–1.46)
CLD	13 (3.9)	7 (4.6)	6 (3.4)	0.56	0.72 (0.23–2.19)
Charlson index	3 [2–5]	3 [2–4]	4 [2–5]	<0.01	1.23 (1.1–1.38)
*Clinical ICU presenting features*					
SAPS II score	39 [30–52]	36 [27–45]	42 [33–57]	<0.01	1.04 (1.03–1.06)
SOFA score	5 [4–7]	4 [3–6]	5 [4–7]	<0.01	1.24 (1.12–1.36)
Pre-ICU Hospital LOS, days	5 [2–8]	4 [2–7]	5 [3–9]	0.025	1.05 (1.01–1.10)
Time from symptoms, days	9 [6–13]	9 [5–12]	10 [6–14]	0.38	1.02 (0.98–1.05)
Septic Shock	74 (22.4)	29 (19.1)	45 (25.1)	0.19	1.42 (0.84–2.4)
Barotrauma	43 (13.0)	23 (15.1)	20 (11.2)	0.29	0.71 (0.37–1.34)
Pulmonary embolism	18 (5.4)	5 (3.3)	13 (7.3)	0.12	2.30 (0.80–6.61)
MDR colonization ****	64 (19.3)	19 (12.5)	45 (25.1)	0.004	2.35 (1.31–4.23)
PaO2/FiO2	88 [75–101]	88 [76–101]	88 [73–101]	0.69	1.00 (0.99–1.01)
Body Temperature, T°	37.0 [36.5–37.6]	37.0 [36.4–37.4]	37.0 [36.5–37.6]	0.27	1.13 (0.91.1.42)
*Laboratory ICU presenting features*					
WBC (10^9^/L)	10.60 [7.65–15.11]	9.50 [6.89–13.05]	11.51 [8.30–16.36]	0.72	1.00 (0.99–1.02)
Neutrophils (10^9^/L)	9.41 [6.55–13.28]	8.39 [6.09–11.67]	10.09 [6.92–14.35]	0.002	1.07 (1.03–1.12)
Lymphocytes (10^9^/L)	0.68 [0.46–0.95]	0.69 [0.47–1.07]	0.65 [0.43–0.90]	0.48	0.99 (0.95–1.02)
PCT, ng/mL	0.20 [0.09–0.42]	0.12 [0.06–0.20]	0.29 [0.14–0.98]	<0.001	39.07 (9.37–162.94)
CRP, mg/L	107.60 [51.7–169.50]	86.40 [45.15–163.15]	128.75 [62.75–180.89]	<0.001	1.005 (1.003–1.008)
Platelets (10^9^/L)	244 [174–312]	251 [186–329]	236 [162–295]	0.02	0.99 (0.99–1.00)
D-Dimer, mg/L	1954 [868–4262]	1472 [727–3735]	2142 [965–4895]	0.94	1.00 (1.00–1.00)
LDH, U/L	399 [319–513]	399 [323–529]	402 [316–508]	0.51	1.00 (1.00–1.00)
Fibrinogen, mg/dL	531 [416–677]	530 [416–687]	535 [414–674]	0.80	1.00 (1.00–1.00)
*Radiology ICU presenting features*					
Brixia score, total	9 [6–13]	8 [5–10]	10 [8–12]	<0.001	1.18 (1.11–1.26)
Monolateral consolidation	114 (34.4)	46 (30.3)	68 (38.0)	0.14	1.41 (0.89–2.23)
Bilateral consolidation	76 (22.9)	28 (18.4)	48 (26.8)	0.07	1.62 (0.96–2.75)
Monolateral effusion	77 (23.3)	32 (21.1)	45 (25.1)	0.38	1.26 (0.75–2.11)
Bilateral effusion	36 (10.9)	16 (10.5)	20 (11.2)	0.85	1.07 (0.53–2.14)
*Treatments at ICU admission*					
Dexamethasone	281 (84.9)	128 (84.2)	153 (85.5)	0.75	1.10 (0.60–2.01)
Remdesivir	160 (48.3)	75 (49.3)	85 (47.5)	0.74	0.93 (0.60–1.43)
IL-6 inhibitors	44 (13.3)	20 (13.2)	24 (13.4)	0.95	1.02 (0.54–1.93)
*Outcomes*					
Post-BAL ICU LOS, days	15 [8–25]	16 [11–30]	13 [6–23]	0.51	0.99 (0.98–1.01)
Post-BAL Hospital LOS, days	20 [9–36]	22 [13–38]	16 [7–35]	0.62	1.00 (0.99–1.01)
Tracheostomy	109 (32.9)	54 (35.5)	55 (30.7)	0.36	0.81 (0.51–1.28)
28-day mortality	144 (43.5)	66 (43.4)	78 (43.6)	0.98	1.01 (0.65–1.56)
90-day mortality	166 (50.3)	77 (51.0)	89 (49.7)	0.82	0.95 (0.62–1.47)

* Previous three months; ** Including coronary and congestive disease; *** Including active neoplasm, chronic steroids, and immunosuppressive agents; **** MRSA nasal colonization and CRE/CRPa/CRAb rectal colonization. Categorical variables are expressed in count and percentage; continuous variables are expressed in median and interquartile range. We included all variables in the multivariable logistic regression if they reached *p* ≤ 0.05 during univariate analysis. A stepwise selection procedure was used to select variables for inclusion in the final model. Legend: CHD: chronic heart disease; COPD: chronic obstructive pulmonary disease; CKD: chronic kidney disease; LOS: length of stay; ICU: intensive care unit; BMI: body mass index; CLD: chronic liver disease; SAPS II: Simplified Acute Physiology Score II; SOFA: Sequential Organ Failure Assessment; MDR: multidrug-resistant; WBC: white blood cells; PCT: procalcitonin; CRP: C-reactive protein; LDH: lactate dehydrogenase; IL-6: interleukin-6; BAL: bronchoalveolar lavage; MRSA: methicillin-resistant *Staphylococcus aureus*; CRE: carbapenem-resistant *Enterobacterales*; CRAb: carbapenem-resistant *Acinetobacter baumannii*; CRPa: carbapenem-resistant *Pseudomonas aeruginosa*.

**Table 2:** Microbiological features and antibiotic therapies of patients with pulmonary co-infection.

Variable	Total (n = 179)
Ongoing empiric antimicrobial therapy	60 (33.5)
Cephalosporins	21 (11.7)
β-lactamase + inhibitors	27 (15.1)
Macrolides	19 (10.6)
Fluoroquinolones	6 (3.4)
Linezolid	6 (3.4)
Vancomycin	5 (2.8)
Carbapenems	4 (2.2)
Other antibiotics *	5 (2.8)
Microbiological data	
Gram-positive cocci	102 (57)
- Methicillin-sensitive *Staphylococcus aureus*	51 (28.5)
- Methicillin-resistant *Staphylococcus aureus*	29 (16.2)
- *Streptococcus pneumoniae*	14 (7.8)
- Other Gram-positive germs **	8 (4.5)
Gram-negative bacilli	113 (63.1)
- *Pseudomonas aeruginosa*	31 (17.3)
- *Klebsiella pneumoniae*	25 (13.9)
- *Haemophilus influenzae*	14 (7.8)
- *Escherichia coli*	9 (5.0)
- Other *Klebsiella* spp.	9 (5.0)
- *Enterobacter cloacae*	7 (3.9)
- *Stenotrophomonas maltophilia*	6 (3.4)
- Other Gram-negative germs ***	12 (6.7)
MDR	36 (20.1)
Polymicrobial ****	42 (23.5)
Concomitant BSI	32 (17.9)

Data are expressed as absolute and relative percentage frequency. * Trimethoprim/sulfamethoxazole (n = 3); Teicoplanin (n = 2). ** *Enteroccus* spp. (n = 5); *Streptoccus agalactiae* (n = 1); *Abiotrophia defectiva* (n = 1); *Streptococcus canis* (n = 1). *** *Serratia marcescens* (n = 5); *Citrobacter koseri* (n = 4); *Moraxella catarrhalis* (n = 2); *Proteus mirabilis* (n = 1). **** Including four cases of fungal–bacterial co-infection (*Aspergillus* spp.). Abbreviations: MDR: multidrug-resistant; BSI: bloodstream Infection.

**Table 3:** Logistic regression analysis of factors associated with bacterial pulmonary co-infection at ICU admission and score development.

Variables	*p* Value	OR (95% CI)	β Value	Risk Score Point
COVID-19 Vaccinated	0.006	7.01 (1.73–28.39)	1.95	2
CKD	0.026	3.16 (1.15–8.71)	1.15	1
Pre-ICU Hospital LOS ≥ 5, days	0.021	1.94 (1.11–3.4)	0.74	1
Neutrophils, ≥9.4 (10^9^/L)	0.013	1.96 (1.16–3.30)	0.67	1
PCT ≥ 0.2 ng/mL	<0.001	5.09 (2.93–8.84)	1.63	2
CRP ≥ 108 mg/L	0.015	1.99 (1.15–3.46)	0.69	1
BRIXIA score ≥ 9	0.009	2.03 (1.19–3.45)	0.71	1

To develop the risk score, variables in the multivariate logistic regression model were assigned a point value corresponding to the β-coefficient (fixed effects) rounded to the nearest integer. The total score was obtained by summation of the individual variables scores. Legend: CKD: chronic kidney disease; LOS: length of stay; ICU: intensive care unit; PCT: procalcitonin; CRP: C-reactive protein.

**Table 4:** Model and risk score performance.

C19-PNEUMOSCORE	TP	FP	TN	FN	Se (%)	Sp (%)	PPV (%)	NPV (%)	Acc (%)	Youden Index
Score ≥ 1	179	144	8	0	100	5.3	55.4	1	56.5	0.05
Score ≥ 2	171	105	47	8	95.5	30.9	62.0	85.5	65.9	0.26
Score ≥ 3	152	64	88	27	84.9	57.9	70.4	76.5	72.5	0.43
Score ≥ 4	122	32	120	57	68.2	78.9	79.2	67.8	73.1	0.47
Score ≥ 5	83	10	142	96	46.4	93.4	89.2	59.7	68.0	0.4
Score ≥ 6	40	2	150	139	22.3	98.7	95.2	51.9	57.4	0.21
Score ≥ 7	13	1	151	166	7.3	99.3	92.9	47.6	49.5	0.07
Score ≥ 8	5	0	152	174	2.8	100	100	46.6	47.4	0.03

Legend: TP: true positive; FP: false positive; TN: true negative; FN: false negative; Se: sensitivity; Sp: specificity: PPV: positive predictive value, NPV: negative predictive value; Acc: accuracy.

## Data Availability

The datasets used and/or analyzed during the current study are available from the corresponding author on reasonable request.
